# Surgical versus medical management of patients with acute ischemic mitral regurgitation: a systematic review

**DOI:** 10.1186/s13104-015-1704-9

**Published:** 2015-11-24

**Authors:** Wissam A. Alajaji, Elie A. Akl, Aida Farha, Wael A. Jaber, Wael A. AlJaroudi

**Affiliations:** Division of Cardiovascular Medicine, American University of Beirut Medical Center, Beirut, Lebanon; Department of Internal Medicine, American University of Beirut Medical Center, Beirut, Lebanon; Department of Clinical Epidemiology and Biostatistics, McMaster University, Hamilton, Canada; Saab Medical Library, American University of Beirut, Beirut, Lebanon; Division of Cardiovascular Medicine, Heart and Vascular Institute, Cleveland Clinic, Cleveland, OH USA; Division of Cardiovascular Medicine/Cardiovascular Imaging, Clemenceau Medical Center, Beirut, Lebanon

**Keywords:** Mitral regurgitation, Acute myocardial infarction, Mitral valve surgery, Mortality

## Abstract

**Aims:**

Acute ischemic mitral regurgitation (MR) is seen in patients with myocardial infarction and is associated with increased morbidity and mortality. The optimal treatment strategy of this condition however, is not well established. The aim of this manuscript is to conduct a systematic review of the medical literature to assess the relative benefits and harms of mitral valve surgery with medical therapy versus medical management alone for patients with acute ischemic MR of at least moderate severity.

**Methods:**

We performed a literature search in MEDLINE, Embase.com, and Cochrane Central Register of Controlled Trials. We restricted the search to randomized clinical trials comparing surgical to medical management of acute ischemic MR. Exclusion criteria included non-randomized trials, trials enrolling patients with non-ischemic MR, and trials excluding acute ischemic MR. The primary outcomes were short-term and long term mortality. Two reviewers (WA, WA) screened titles and abstracts of identified citations independently and in duplicate using calibration exercises and standardized screening forms.

**Results:**

The search strategy identified 887 citations (137 were duplicates and removed). Of the 750 titles, 709 were excluded (519 were non-relevant and 190 were review articles and case reports). Of the 41 remaining abstracts, 37 were retrospective cohorts and four excluded acute MR, leaving no eligible study for analysis. An ongoing study that is being conducted at Southern Illinois University entitled by “Medical Versus Surgical Management of Patients With Moderate Mitral Regurgitation Following Percutaneous Coronary Intervention for Myocardial Infarction: A Pilot Prospective Randomized Trial” was identified; however, it was just withdrawn after failing to enroll patients during 4 years.

**Conclusion:**

This is an empty systematic review that identified no published randomized trials for the management of acute MR complicating acute MI. The only ongoing randomized study that was identified was just withdrawn after failing to enroll patients. There is an urgent need for conducing proper randomized trials in order to guide informed decision making in the treatment of acute ischemic MR.

PROSPERO registration number CRD42013005843

**Electronic supplementary material:**

The online version of this article (doi:10.1186/s13104-015-1704-9) contains supplementary material, which is available to authorized users.

## Background

Acute ischemic mitral regurgitation (MR) is seen in patients with myocardial infarction (MI), and is associated with increased morbidity and mortality. Severe MR may occur with a frequency ranging from 0.3 to 3 % [1, 2] and is more related to the location of the infarction (more commonly seen with inferior MI) rather than the degree of infarction [3–7]. Left ventricular remodeling, tethering of the mitral valve leaflets, apical displacement of the papillary muscle, and less commonly papillary muscle rupture, contribute to the MR [2, 8–10]. It is generally accepted that patients with acute ischemic MR due to papillary muscle rupture need emergent mitral valve surgery given the exceedingly high mortality rate (up to 80 %) without intervention [11, 12]. However, in patients with acute ischemic MR but without papillary muscle rupture, there is no general consensus whether mitral valve surgery is warranted or beneficial as opposed to medical therapy alone. Subgroup analysis of the SHOCK registry (Should we revascularize Occluded Coronaries for cardiogenic shocK) showed that early mitral valve replacement was associated with better survival when compared to medical therapy alone [6]. However, these were observational data with selection bias. Hence, the aim of this study was to conduct a systematic review of the medical literature to assess the relative benefits and harms of mitral valve surgery with medical therapy versus medical therapy alone for patients with acute ischemic MR.

## Methods

We published the research protocol of this systematic review online on PROSPERO systematic review database prior to the literature search and data extraction. There were no modifications between the planned protocol and the actual conduct of the review.

Reference URL for published protocol: http://www.crd.york.ac.uk/PROSPERO/display_record.asp?ID=CRD42013005843. Search strategy: http://www.crd.york.ac.uk/PROSPEROFILES/5843_PROTOCOL_20131106.pdf. (PROSPERO registration number CRD42013005843).

### Eligibility

Inclusion criteria: The systematic review was designed to include only randomized controlled trials. Exclusion criteria: were non-randomized trials, trials enrolling patients with non-ischemic MR such as rheumatic or endocarditis, or and trials excluding acute ischemic MR. Population: Enrolling patients with acute ischemic MR of at least moderate severity, and Intervention: comparing control: medical management versus mitral valve surgery (annuloplasty, repair, or replacement strategy) with respect to mortality outcome. Outcome: The pre-defined primary outcomes were short-term and long-term mortality. The secondary outcomes were surgical complications, need for surgical intervention, recurrent MI, stroke, heart failure, quality of life, and/or length of hospitalization.

### Literature search

We conducted a literature search in MEDLINE, Embase.com, and Cochrane Central Register of Controlled Trials (CENTRAL). The searches were performed in Cochrane on September, 26, 2013; Medline and Embase.com searches were conducted on November, 21, 2013. We did not apply language or publication period restrictions. Additional file 1: Appendix S1 shows the search strategies. We also searched for ongoing research trials about the subject in http://www.controlled-trials.com/isrctn/ and http://www.clinicaltrials.gov.

The search strategy was drafted by two of the co-authors (WA, EA), one of whom (EA) has experience in designing search strategies. An information specialist with experience in systematic reviews (Ms. Aida Farha, head librarian) revised and refined the strategy. We did not subject the search strategy to external peer review [13].

### Selection, data abstraction, and risk of bias assessment

Two reviewers (WA, WA) screened the titles and abstracts of identified citations independently and in duplicate. The full texts for citations that were judged by at least one of the two reviewers as potentially eligible were subsequently retrieved and screened independently. The results of the screening process were compared, and any disagreement was resolved by either discussion or with the help of a third reviewer. Calibration exercises were conducted and standardized screening forms based on the eligibility criteria were used. We planned for a similar approach for data abstraction. We planned to assess the risk of bias in each study independently and in duplicate using the Cochrane Risk of Bias tool.

### Strategy for data synthesis

We assessed the agreement between the two authors for the assessment of trial eligibility using kappa statistic. Our protocol details the planned analysis plan that we did not have the opportunity to use.

## Results

The search strategy identified 887 citations (137 were duplicates and removed). Of the 750 titles, 709 were excluded (519 were non-relevant and 190 were review articles and case reports). Of the 41 remaining abstracts, 37 were retrospective cohorts and four excluded acute MR, leaving no eligible study for analysis (Additional file 2: Figure S1). Based on our search strategy, we did not find any published randomized controlled trial comparing outcomes of medical versus surgical treatment of acute ischemic MR. However, we found one registered ongoing randomized controlled trial that is being conducted at Southern Illinois University entitled by “Medical Versus Surgical Management of Patients With Moderate Mitral Regurgitation Following Percutaneous Coronary Intervention for Myocardial Infarction: A Pilot Prospective Randomized Trial” (ClinicalTrials.gov Identifier: NCT01156441). Unfortunately, the study has been just withdrawn in June 2014 with no enrollment after 4 years.

## Discussion

Our systematic review serves as the first one in the medical literature that attempts to summarize the evidence from randomized controlled trials comparing medical to surgical management of at least acute moderate ischemic MR. We found no published randomized controlled clinical trials to answer our inquiry.

The current review is an empty review given no studies met the eligibility criteria. Empty reviews are not uncommon and account for 8.7–12 % of Cochrane database of systematic reviews [14, 15]. One reason for this review being empty is that we limited inclusion to randomized clinical trials. The intention was to exclude data from non-randomized studies given they are unlikely to be reasonably informative for either guideline development or clinical decisions.

Indeed, the available non-randomized data on acute ischemic MR are based on case reports, case series, and registry analyses (Table 1) [6, 9, 16–21]. Typically in these studies, patients with higher ejection fraction received mitral valve intervention significantly more often than patients with lower ejection fraction. This selection bias is likely to impact the effect estimates of the outcome of interest. As a result, the confidence in the effect estimates (i.e., quality of evidence) would be at best low [22]. Also, higher quality evidence was expected to be available with the publication of the ongoing randomized trial (ClinicalTrials.gov Identifier: NCT01156441); unfortunately the study has been just withdrawn in June 2014 with no enrollment after 4 years.

**Table 1:** Summary and pooled analysis of non-randomized studies of patients presenting with acute ischemic mitral regurgitation

Study	Study type	Year	N	Age (years)	NYHA III–IV (%)	EF %	<30 day mortality (N)		1–12 months mortality^a^ (N)		1–15 year mortality^a^ (N)	
							MVR	No MVR	MVR	No MVR	MVR	No MVR
Nishimura [16]	Case series	1986	7	57	100	49	0/7	(–)	3/7	(–)	1/7	(–)
Kishon [17]^b^	Retrospective	1992	22	68			6/22	(–)	(–)	(–)	6/22	(–)
Tcheng [9]	Retrospective	1992	50	68		35	(–)	16/50	(–)	25/50	(–)	(–)
Gillinov [18]	Retrospective	2001	95				7/95	(–)	6/95	(–)	74/95	(–)
Minami [19]^b^	Retrospective	2004	6		100	57	2/6	(–)	(–)	(–)	(–)	(–)
Russo [20]^b^	Prospective	2008	54	70	98	56	2/54	(–)	(–)	(–)	(–)	(–)
Lorusso [21]^b^	Retrospective	2008	126	62	66	#	34/126	(–)	(–)	(–)	8/126	(–)
Thompson [6]	Prospective	2009	94	71	100	37	17/43	36/51	(–)	(–)	(–)	(–)
Pooled			454	67	91	42	68/353	52/101	9/102	25/50	89/252	(–)

*EF* ejection fraction, *MVR* mitral valve repair or replacement, *N* number of patients with acute ischemic mitral regurgitation, *NYHA* New York Heart Association

^#^Half of patients had left ventricular systolic dysfunction; (–), not applicable or no patients enrolled in that arm

^a^These is not cumulative mortality. To get cumulative mortality, add up the numbers where applicable

^b^Mixed studies including patients with papillary muscle rupture

Importantly, our systematic review shows the gap in the knowledge about what is the best intervention in the treatment of MR following acute myocardial infarction. It is our hope that this empty systematic review will help stimulate appropriate research, whether well-designed prospective observational studies or, ideally, well-designed and adequately powered, randomized clinical trials.

There were several retrospective data on the subject as summarized in Table 1. Of the observational data that we encountered, one was an analysis of the SHOCK registry comparing mortality outcome of severe acute ischemic MR treated by mitral valve annuloplasty repair/replacement versus no valve intervention [6]. Between April 1993 and August 1997, the SHOCK registry enrolled 1190 patients with suspected cardiogenic shock complicating acute MI. A subgroup analysis of this registry included 94 patients with severe MR. Almost half of the cohort (N = 43) underwent concomitant coronary artery bypass graft with mitral valve surgery (valve replacement was done in most and valve repair was done in six patients only), while the remaining patients (N = 51) underwent coronary artery bypass grafting alone with medical management of the MR. The in-hospital mortality rates for valve surgery and non-valve surgery groups were 40 and 71 % respectively [6]. The lower mortality in the group that underwent valve surgery however, was predominately driven by a significantly higher left ventricular ejection fraction (40 versus 29 %, p = 0.04), causing a selection bias. These observational data that were identified non-systematically showed some potential benefit for early surgery given the high mortality rate of un-intervened severe MR following acute MI [6]. These results must be interpreted with caution however Another multicenter retrospective study evaluated the postoperative outcome of emergency surgery for acute severe MR [21]. Of 279 total patients enrolled, 126 (45 %) had an acute MI and did worse than those without MI. On multivariable analysis, associated coronary artery disease and acute MI were independent predictors of increased short-term and long-term mortality. The type of mitral valve surgery (repair versus replacement), however did not influence the outcomes [21].

When looking at the data of the non-randomized studies (Table 1), one could notice the mixed cohorts that included patients with papillary muscle rupture versus leaflet tethering. These are totally different cohorts; yet, the reported outcomes are combined and pooled for all patients. Furthermore, the studies were of small sample size, most of them involving only one treatment arm (most often mitral valve replacement) which makes any possibility of fair comparison of treatment strategy to outcomes almost impossible. In addition, the cohorts included a very high prevalence of patients with cardiogenic shock and already depressed left ventricular systolic function. Pooling the numbers of all the studies did show a significantly lower in-hospital (<30 days) and 1–12 months mortality for the registry of patients that underwent MVR (68/353 versus 51/101 for in-hospital mortality, and 9/102 versus 25/50 for 1–12 months mortality, respectively) (Table 1). However these numbers must be interpreted very cautiously due to the inhomogeneity of the cohorts, study designs and selection bias.

## Implications for practice

**Fig. 1:**
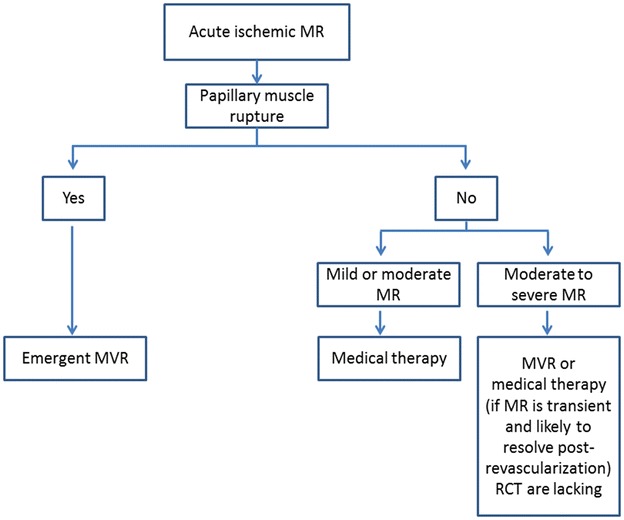
Proposed algorithm for management of patients with acute ischemic mitral regurgitation. *MR* mitral regurgitation; *MVR* mitral valve replacement/repair; *RCT* randomized clinical trial

In absence of robust data, the management of acute ischemic MR mostly remains based on expert opinion. Nowadays at least, the standards of management involve the early identification of MR during acute MI, and the differentiation of whether there is papillary or chordal disruption or not. It is clinically relevant to differentiate papillary muscle ischemia from papillary muscle/chordal rupture, since the former might be corrected by revascularization alone, hence sparing the patient the added risk of mitral valve repair or replacement. Indeed, observational data suggested that acute ischemic MR without papillary muscle rupture may be treated with coronary angioplasty; the 3 year survival of coronary intervention group was 70.2 versus 45.6 % in medical group [23]. Figure 1 is a proposed algorithm that summarizes a potential approach in the management of patients with acute ischemic MR.

Papillary muscle rupture, on the other hand, is an unusual cause of ischemic MR occurring in less than 0.1 % of patients [24]. When present, it carries a poor prognosis with mortality rates reaching 95 % following MI if left without surgical intervention, and with 70 % of death occurring within the first 24 h [11, 25]. Other observational data studies showed that the mortality rate is high regardless of valve surgery; it appears to be that these patients are at an already advanced stage where intervention may be too late whenever shock occurs [7]. Data from a larger and more recent study, however, showed that there has been considerable decrease in perioperative mortality across decades (after 1990 as compared to prior to 1990) for patients with acute MR secondary to papillary muscle rupture undergoing mitral valve surgery, particularly when concomitant coronary artery bypass graft is performed (odds ratio 0.18; 95 % confidence interval 0.04–0.83) [20]. Also, the long term survival was similar to those who had an MI without papillary muscle rupture [20].

Furthermore, Chevalier et al. evaluated the predictors of increased perioperative mortality of 55 consecutive patients with acute MR (45 % papillary muscle rupture, 22 % partial rupture, and 33 % papillary muscle dysfunction) post MI undergoing mitral valve surgery (94 % valve replacement). Although the perioperative mortality was high, it was significantly reduced when concomitant coronary artery bypass graft was performed (34 versus 9 %, p = 0.02) [26].

In patients presenting with acute ischemic MR, those with papillary muscle rupture warrant emergent mitral valve repair/replacement, while those with papillary muscle dysfunction or leaflet tethering might have significant improvement in the degree of MR after coronary revascularization and optimal medical therapy (Fig. 1). The role of intra-operative transesophageal echocardiogram in guiding whether MVR is needed as an adjunct to coronary revascularization in such situation seems justified, although there is no consensus on such approach that varies with different clinical practices.

### Limitations

A major limitation is the lack of RCT ending up with an empty systematic review. Furthermore, we have lumped several types of acute ischemic MR (such as papillary muscle rupture, apical tethering) in the search. While these subtypes of acute MR behave completely and require a different therapeutic approach and therefore cannot be lumped together, we opted to include any kind of acute ischemic MR in the search to avoid exclusion of studies with such subgroups. It was our intention to analyze the outcomes of each type of acute MR differently; unfortunately, there were no RCT that met our inclusion criteria to start with. Furthermore, it was kind of expected to have limited RCT given the borderline ethical justification one might raise in performing such randomized study. While attempting to do pooled analysis of the non-RCT that were found, we were limited by the heterogeneity of the outcomes and cohorts that prevented any meaningful large scale data pooling.

## Conclusion

Based on our systematic review we conclude that there is a lack of randomized data comparing the clinical outcomes of medical management versus mitral valve surgery in patients with acute MR of at least moderate severity. In absence of randomized controlled trials, it is hard to make a solid informed decision about the optimal management of acute severe ischemic MR in the absence of papillary muscle rupture, and the treatment remains to be based on expert opinion and physician expertise. Medical management in such situation might be reasonable if the degree of MR is less than severe or if there high suspicion of improvement and reversibility of the MR with coronary revascularization. The withdrawal of the randomized trial “Medical Versus Surgical Management of Patients With Moderate Mitral Regurgitation Following Percutaneous Coronary Intervention for Myocardial Infarction” due to failure to enroll patients was disappointing. It is our hope that this empty systematic review will help stimulate appropriate research, whether well-designed prospective observational studies or, ideally, well-designed and adequately powered, randomized clinical trials.

## Additional files

10.1186/s13104-015-1704-9 Search strategy.
10.1186/s13104-015-1704-9 Prisma 2009 flow diagram.

## Abbreviations

MI: myocardial infarction
MR: mitral regurgitation
SHOCK: Should we revascularize Occluded Coronaries for cardiogenic shocK
