# Supplementary material for: Surgical versus medical management of patients with acute ischemic mitral regurgitation: a systematic review
Source: BMC Res Notes. 2015 Nov 24;8:712. doi: 10.1186/s13104-015-1704-9 (PMC4659221; doi:10.1186/s13104-015-1704-9)
Supplement: Supplementary file 1 — 10.1186/s13104-015-1704-9 Search strategy. [file 13104_2015_1704_MOESM1_ESM.docx]

**Appendix S1:**

Database: Ovid MEDLINE(R) <1946 to November Week 3 2013>

Search Strategy:

--------------------------------------------------------------------------------

1 exp Mitral Valve Insufficiency/ (17712)

2 exp Heart Valve Diseases/ (90781)

3 exp Mitral Valve/ (22797)

4 mitral.mp. (63650)

5 2 and (3 or 4) (40148)

6 exp Papillary Muscles/ (7237)

7 exp Chordae Tendineae/ (1612)

8 (chorda* adj3 Tendin*).mp. [mp=title, abstract, original title, name of substance

word, subject heading word, keyword heading word, protocol supplementary concept,

rare disease supplementary concept, unique identifier] (2101)

9 (8 or 6 or 7) and (3 or 4) (2755)

10 (mitral adj3 (regurg* or chord* or incompet* or flail* or papill* or tether* or leak*

or ruptur* or malcoapt* or prolaps* or insufficien* or perforat* or enlarge*)).mp.

[mp=title, abstract, original title, name of substance word, subject heading word,

keyword heading word, protocol supplementary concept, rare disease supplementary

concept, unique identifier] (26715)

11 1 or 5 or 9 or 10 (44220)

12 exp myocardial ischemia/ or exp acute coronary syndrome/ or exp angina

pectoris/ or exp angina, unstable/ or exp angina pectoris, variant/ or exp coronary

disease/ or exp coronary artery disease/ or exp coronary occlusion/ or exp coronary

stenosis/ or exp coronary restenosis/ or exp coronary thrombosis/ or exp coronary

vasospasm/ or exp myocardial infarction/ or exp anterior wall myocardial infarction/ or

exp inferior wall myocardial infarction/ or exp myocardial stunning/ or exp shock,

cardiogenic/ or exp myocardial reperfusion injury/ (359420)

13 (heart adj3 (infarct* or attack* or injur* or ischemi* or ischaemi* or thromb* or

atherosclero*)).mp. [mp=title, abstract, original title, name of substance word, subject

heading word, keyword heading word, protocol supplementary concept, rare disease

supplementary concept, unique identifier] (54207)

14 (coronary adj3 (occlu* or steno* or ischemi* or ischaemi* or shock* or thromb*

or event* or syndrome* or vasospas* or unstable* or attack* or insufficien* or

resteno*)).mp. [mp=title, abstract, original title, name of substance word, subject

heading word, keyword heading word, protocol supplementary concept, rare disease

supplementary concept, unique identifier] (75954)

15 (STEMI or NSTEMI or UA or MI or unstable angina*).ti,ab. (45012)

16 (myocardi* adj3 (infarct* or ischemi* or ischaemi* or necro* or stun* or injur* or

reperfus* or attack* or dysfunction*)).mp. [mp=title, abstract, original title, name of

substance word, subject heading word, keyword heading word, protocol

supplementary concept, rare disease supplementary concept, unique identifier]

(244355)

17 12 or 13 or 14 or 15 or 16 (440255)

18 exp Mitral Valve Annuloplasty/ (413)

19 exp Heart Valve Prosthesis/ (28005)

20 exp Heart Valve Prosthesis Implantation/ (12963)

21 exp Cardiac Surgical Procedures/ (167028)

22 (Mitral adj4 (prosthe* or insert* or implant* or repair* or replac* or plac* or

annuloplast* or surg* or interven* or Procedure* or clip*)).mp. [mp=title, abstract,

original title, name of substance word, subject heading word, keyword heading word,

protocol supplementary concept, rare disease supplementary concept, unique

identifier] (16355)

23 18 or 19 or 20 or 21 or 22 (194104)

24 (randomized controlled trial or controlled clinical trial).pt. or randomized.ab. or

placebo.ab. or drug therapy.fs. or randomly.ab. or trial.ab. or groups.ab. (3308511)

25 exp animals/ not humans.sh. (4066609)

26 24 not 25 (2817704)

27 11 and 17 and 23 and 26 (440)

***************************

Embase.com

No. Query Results

#1 383

#1.49 #1.15 AND #1.23 AND #1.39 AND #1.48 383

#1.48 #1.40 OR #1.43 OR #1.44 OR #1.45 OR #1.46 OR #1.47

#1.47 mitral NEAR/4 (regurg* OR chord* OR incompet* OR flail* OR papill* OR tether* OR leak* OR ruptur* OR malcoapt* OR prolaps* OR insufficien* OR perforat* OR enlarge*)

#1.46 'mitral regurgitation'/exp OR mitral AND regurgitati*

#1.45 'chordae tendineae rupture'/exp OR chordae AND tendineae AND ruptur*

#1.44 'papillary muscles'/exp OR papillary AND muscle*

#1.43 #1.41 AND #1.42

#1.42 mitral AND ('valve'/exp OR valve*)

#1.41 'heart valve diseases'/exp OR heart AND valve AND disease*

#1.40 'mitral valve insufficiency'/exp OR mitral NEAR/4 insufficien*

#1.39 #1.24 OR #1.25 OR #1.26 OR #1.27 OR #1.28 OR #1.29 OR #1.30 OR #1.31 OR #1.32 OR #1.33 OR #1.34 OR #1.35 OR #1.36 OR #1.37 OR #1.38

#1.38 'acute coronary syndrome'/exp OR acute AND coronary AND syndrome*

#1.37 'unstable angina'/exp OR unstable AND angina*

#1.36 'acute coronary artery thrombosis'/exp OR acute AND coronary AND thrombo*

#1.35 'st elevated myocardial infarction'/exp OR st AND elevat* AND myocardial AND infarct*

#1.34 non AND ('st'/exp OR st) AND segment AND elevat*

#1.33 'non st elevated myocardial infarction'/exp OR non AND st AND elevat* AND myocardial AND infarct*

#1.32 'st elevation'/exp OR 'st elevation'

#1.31 myocardi* NEAR/4 (infarct* OR ischemi* OR ischaemi* OR necro* OR stun* OR injur* OR reperfus* OR attack* OR dysfunction*)

#1.30 coronary NEAR/4 (occlu* OR steno* OR ischemi* OR ischaemi* OR shock* OR thromb* OR event* OR syndrome* OR vasospas* OR unstable* OR attack* OR insufficien* OR resteno*)

#1.29 'angina pectoris'/exp OR 'angina pectoris'

#1.28 heart NEAR/4 (infarct* OR attack* OR injur* OR ischemi* OR ischaemi* OR thromb* OR atherosclero*)

#1.27 'anteroseptal myocardial infarction'/exp OR anteroseptal AND myocardial AND infarct*

#1.26 'inferior wall myocardial infarction'/exp OR inferior AND wall AND myocardial AND infarct*

#1.25 'ischemic mitral regurgitation'

#1.24 'cardiogenic shock'/exp OR cardiogenic AND shock*

#1.23 #1.16 OR #1.17 OR #1.18 OR #1.19 OR #1.20 OR #1.21 OR #1.22

#1.22 mitral NEAR/4 (prosthe* OR insert* OR implant* OR repair* OR replac* OR plac* OR annuloplast* OR surg* OR interven* OR procedure* OR clip*)

#1.21 'heart valve bioprosthesis'/exp OR 'heart valve bioprosthesis'

#1.20 'mitral valve prosthesis'/exp OR 'mitral valve prosthesis'

#1.19 'mitral valve replacement'/exp OR 'mitral valve replacement'

#1.18 'mitral valve repair'/exp OR 'mitral valve repair'

#1.17 'mitral annuloplasty'/exp OR 'mitral annuloplasty'

#1.16 'mitral valvuloplasty'/exp OR 'mitral valvuloplasty'

#1.15 #1.1 OR #1.2 OR #1.3 OR #1.4 OR #1.5 OR #1.6 OR #1.7 OR #1.8 OR #1.9 OR #1.10 OR #1.11 OR #1.12 OR #1.13 OR #1.14

#1.14 random*

#1.13 factorial*

#1.12 crossover*

#1.11 cross AND over*

#1.10 placebo*

#1.9 doubl* NEAR/2 blind*

#1.8 singl* NEAR/2 blind*

#1.7 assign*

#1.6 allocat*

#1.5 volunteer*

#1.4 'crossover procedure'/exp OR 'crossover procedure'

#1.3 'double blind' AND ('procedure'/exp OR procedure)

#1.2 randomized AND controlled AND trial

#1.1 'single blind' AND ('procedure'/exp OR procedure)

***************************

Database: CENTRAL Search Name: final strategy

Last Saved: 26/09/2013 18:17:20.329

Description:

ID Search

#1 MeSH descriptor: [Mitral Valve Annuloplasty] explode all trees

#2 MeSH descriptor: [Cardiac Valve Annuloplasty] explode all trees

#3 MeSH descriptor: [Heart Valve Prosthesis] explode all trees

#4 mitral near (replac* or surg* or interven* or implant* or annuloplast* or prosthe* or repair* or clip* or place* or insert*)

#5 #1 or #2 or #3 or #4

#6 MeSH descriptor: [Acute Coronary Syndrome] explode all trees

#7 MeSH descriptor: [Myocardial Ischemia] explode all trees

#8 MeSH descriptor: [Angina Pectoris] explode all trees

#9 MeSH descriptor: [Myocardial Infarction] explode all trees

#10 MeSH descriptor: [Myocardial Reperfusion Injury] explode all trees

#11 MeSH descriptor: [Myocardial Stunning] explode all trees

#12 MeSH descriptor: [Shock, Cardiogenic] explode all trees

#13 MeSH descriptor: [Myocardial Stunning] explode all trees

#14 MeSH descriptor: [Anterior Wall Myocardial Infarction] explode all trees

#15 MeSH descriptor: [Inferior Wall Myocardial Infarction] explode all trees

#16 MeSH descriptor: [Angina, Unstable] explode all trees

#17 myocari* near (ischemi* or ischaemi* or infarct* or attack* or event* or dysfunction* or necro* or shock*)

#18 coronary near (occlu* or thomb* or insufficien* or event* or attack* or ischemi* or ischaemi* or steno* or narrow* or plaque ruptur* or plaque ulcer* or plaque fissur* or acute syndrom* or unstable* or infarct*)

#19 #6 or #7 or #8 or #9 or #10 or #11 or #12 or #13 or #14 or #15 or #16 or #17 or #18

#20 Heart Valve Disease*

#21 heart valve disease

#22 mitral near (insufficien* or incompeten* or leak* or prolaps* or perfora* or ruptur* or regurgitat* or flail* or malcoaptat* or noncoaptat* or malfunction* or dysfunction*)

#23 MeSH descriptor: [Papillary Muscles] explode all trees

#24 MeSH descriptor: [Chordae Tendineae] explode all trees

#25 papillary near (ruptur* or dysfunction* or malfunction*)

#26 chordae* near (ruptur* or dysfunction* or malfunction*)

#27 #20 or #21 or #22 or #23 or #24 or #25 or #26

#28 #5 and #19 and #27
