# Supplementary material for: Surgical versus medical management of patients with acute ischemic mitral regurgitation: a systematic review
Source: BMC Res Notes. 2015 Nov 24;8:712. doi: 10.1186/s13104-015-1704-9 (PMC4659221; doi:10.1186/s13104-015-1704-9)
Supplement: Supplementary file 2 — 10.1186/s13104-015-1704-9 Prisma 2009 flow diagram. [file 13104_2015_1704_MOESM2_ESM.doc]

**Figure S1.**


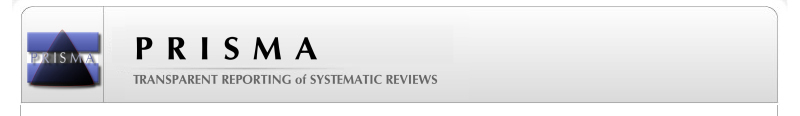
**PRISMA 2009 Flow Diagram**

**Screening**

**Included**

**Eligibility**

**Identification**

Records identified through database and all sources searching
(n = 887 )

Records after duplicates removed
(n =750 )

Records screened
(n =750 )

Records excluded
(n =709 )

1. 519 Non-relevant
2. 190Review articles and case reports

Full-text articles assessed for eligibility
(n =41)

Full-text articles excluded, with reasons
(n =41 )

1. 37 retrospective cohort
2. 4 Excluded Acute MR

Studies included in qualitative synthesis/eligible papers
(n =0 )
